# Call for a Convention of Physicians

**Published:** 1871-06

**Authors:** 


### CALL FOR A CONVENTION OF PHYSICIANS.

It has been proposed that the profession of the State signify their assent to the proposition of having a Convention of the Physicians of the State by signing a call. We hope the physicians in every portion of the State will forward their names to W. Duncan, M. D., Savannah, Ga., approving the call, and signing their names thereto, to meet at Macon, Ga., on the 5th of July next. The object of the Convention is to arrive at the whole truth of the difficulties between the old Faculty of the Atlanta Medical College and the Georgia Medical Association, that the honor of the latter may be untarnished. The movement is intended to embrace all the regular physicians of the State, that they may voluntarily assemble, or authorize the assembling of a convention to give expression to the true professional sentiments of the physicians of the State on the questions upon which it has been the misfortune of the profession, to differ, with the hope of arriving at an amicable and harmonious solution of all matters of controversy. We entreat all true men of the profession to at once forward their names, calling upon the profession to assemble for the purposes contemplated. Friends, do not delay; your interests as professional gentlemen and the honor of the profession demands immediate action. Let every friend of peace and harmony at once forward their names.

